# Winter Hibernation and UCHL1-p34^cdc2^ Association in Toad Oocyte Maturation Competence

**DOI:** 10.1371/journal.pone.0078785

**Published:** 2013-10-23

**Authors:** Zhichao Kuang, Yuwei Yao, Yan Shi, Zheng Gu, Zhaogui Sun, Jiake Tso

**Affiliations:** 1 Institute of Reproduction & Development, Shanghai Medical College, Fudan University, Shanghai, China; 2 Key Laboratory of Contraceptive Drugs and Devices of National Population and Family Planning Commission of China, Shanghai Institute of Planned Parenthood Research, Shanghai, China; Institute of Zoology, Chinese Academy of Sciences, China

## Abstract

Currently, it is believed that toad oocyte maturation is dependent on the physiological conditions of winter hibernation. Previous antibody-blocking experiments have demonstrated that toad ubiquitin carboxyl-terminal hydrolase L1 (tUCHL1) is necessary for germinal vesicle breakdown during toad oocyte maturation. In this paper, we first supply evidence that tUCHL1 is highly evolutionarily conserved. Then, we exclude protein availability and ubiquitin carboxyl-terminal hydrolase enzyme activity as factors in the response of oocytes to winter hibernation. In the context of MPF (maturation promoting factor) controlling oocyte maturation and to further understand the role of UCHL1 in oocyte maturation, we performed adsorption and co-immunoprecipitation experiments using toad oocyte protein extracts and determined that tUCHL1 is associated with MPF in toad oocytes. Recombinant tUCHL1 absorbed p34^cdc2^, a component of MPF, in obviously larger quantities from mature oocytes than from immature oocytes, and p13^suc1^ was isolated from tUCHL1 with a dependence on the ATP regeneration system, suggesting that still other functions may be involved in their association that require phosphorylation. In oocytes from hibernation-interrupted toads, the p34^cdc2^ protein level was significantly lower than in oocytes from toads in artificial hibernation, providing an explanation for the different quantities isolated by recombinant tUCHL1 pull-down and, more importantly, identifying a mechanism involved in the toad oocyte’s dependence on a low environmental temperature during winter hibernation. Therefore, in toads, tUCHL1 binds p34^cdc2^ and plays a role in oocyte maturation. However, neither tUCHL1 nor cyclin B1 respond to low temperatures to facilitate oocyte maturation competence during winter hibernation.

## Introduction

Chinese toads, *Bufo bufo gargarizans*, usually lay eggs in February every year. The small oocytes remain in the ovaries over the summer and grow to their full size during October and November, reaching a diameter of up to approximately 1.8 mm. Then, the toads enter hibernation in response to low temperatures [1]. Hibernating toads ovulate in response to stimulation with pituitary extracts *in vivo*, and the artificially separated oocytes can then be induced to mature by progesterone exposure *in vitro*; however, fully grown oocytes obtained with the hibernation interruption method will not undergo GVBD (germinal vesicle breakdown) under progesterone stimulation [1]. If non-hibernated female toads are artificially maintained at 4 °C for 4-6 weeks, oocytes from those females will have progesterone sensitivity for maturation recovery [2]. When toads captured during November and December are maintained at 4 °C for at least one month, the oocytes achieve maturation competence. These oocytes, subjected to a low-temperature environment treatment, are hereafter referred to as LTE-oocytes (Low-temperature environment-oocytes). In contrast, when the toads are raised in a 28 °C environment for 2-3 months, their oocytes lose the capacity to undergo GVBD in response to progesterone. Such oocytes are hereafter referred to as HTE-oocytes (High-temperature environment-oocytes). With the help of oocyte cytoplasm transplantation, it was determined that LTE-oocyte cytoplasm could remedy the defective maturation competence of HTE-oocytes, but the auto-amplification capability to promote oocyte maturation had been lost in the HTE-oocytes [3]. On the other hand, in another study, HTE-oocyte cytoplasm could not inhibit progesterone-induced maturation in LTE-oocytes [3]. It was further confirmed that H1 kinase activity is not produced in HTE-oocytes [4]. However, whether the MPF constituent (p34^cdc2^ and cyclin B1/B2) synthesis remains unchanged in the HTE-oocytes still requires further confirmation. In addition, if the p34^cdc2^ and cyclin B1/B2 protein content in oocytes is not affected by the high-temperature (winter hibernation interruption) treatment, then the question is raised about what factors in HTE-oocytes are involved in blocking GVBD in response to progesterone stimulation.

Using a pull-down analysis of toad oocyte protein extracts with agarose-conjugated p13^suc1^ beads, a MPF-associated candidate was identified. A much greater quantity of this candidate was absorbed from LTE-oocytes than from HTE-oocytes [4]. A candidate-reactive monoclonal antibody was prepared by cutting out its corresponding band in SDS/PAGE gels as an antigen, and its cDNA sequence was obtained by immunological screening from a cDNA library [5]. Its amino acid residue ratio was 55% identical to human protein gene product 9.5 (PGP9.5) protein. PGP9.5, recently renamed ubiquitin carboxyl-terminal hydrolase (UCHL1) because of its enzyme activity [6,7], is specifically expressed in the neurons, testes, ovaries, and placenta, suggesting it has particular roles in these tissue types [8]. To identify the protein extracted from the toad oocytes, we designated the homolog found in toads as tUCHL1. In our previous studies, we observed that the monoclonal antibody against tUCHL1 could specifically inhibit GVBD, indicating that tUCHL1 is required for oocyte maturation in toads [5]. However, GVBD was inhibited independently of its UCH enzyme activity [9].

In this study, we first determined whether tUCHL1 exhibits a conserved tissue distribution pattern similar to that found for UCHL1 in mammals. Then, we inspected the differences in tUCHL1 between LTE-oocytes and HTE-oocytes and attempted to probe its association with MPF to clarify its role in the dependence of oocyte maturation potential on winter hibernation. 

## Materials and Methods

### Toad animal models and oocyte maturation *in vitro*

Chinese toads (Bufo bufo gargarizans) were collected in the suburb of Jinan City, Shandong Province in October. They were divided into two groups: toads in the artificially hibernated group were maintained at 4 °C for 2-3 months after collection to facilitate development of the oocytes in their ovaries (reach stage IV) to the point of obtaining maturation competence under progesterone stimulation (10-6 mol/L) [4]. The oocytes from the artificially hibernated group are referred to as LTE-oocytes as noted above. For the other group with artificially interrupted hibernation, toads were raised at 28 °C with yellow mealworms (*Tenebrio molitor*) as food for 2-3 months. The oocytes from the artificially interrupted hibernation group are referred to as HTE-oocytes as noted above. After the toads were deeply anesthetized in a MS 222 (tricaine methanesulfonate, Sandoz Ltd.) solution diluted 1/1000 for 15 min, the ovaries were separated for oocyte harvest. Tissue pieces of the ovaries were digested to remove the ovarian theca and follicular cells, exposing the denuded oocytes [4]. The oocytes were examined with GVBD incidence being used as the index of oocyte maturation competence[5]. All the procedures for toad indoor feeding, experiments, and sacrifice methods conformed to the regulations of animal experiment management established by the Shanghai Institute of Planned Parenthood Research and were approved by the animal ethics committee (NO 200801).

### Preparation of protein extracts from various tissues and oocytes

Oocytes and various tissues, including the brain, skin, spermary, stomach, liver, intestine, kidney, muscle, lung, and heart were separated, and 100 mg samples were collected. Each tissue sample was mixed with 500 μl of extraction buffer (50 mmol/L Tris-HCl, pH 7.4, supplemented with 75 mmol/L KCl, 10 mmol/L Na2HPO4·12H2O, 1 mmol/L EDTA·2Na·2H2O, 5 mmol/L NaF, 250 mmol/L sucrose, 10 mmol/L ATP·2Na, 100 μmol/L PMSF, and 10 μg/ml each of soybean trypsin inhibitor (Sigma), leupeptin and aprotinin (Amresco)) and homogenized in Teflon glass homogenizers. The homogenates were centrifuged for 20 min at 4 °C and 36,000 g in a Hitachi CR22F centrifuge (Hitachi, Japan). The transparent supernatants were recovered using pipettes with fine tips to avoid contamination with the upper lipid layer and then centrifuged once more. The supernatants from the second centrifuging were gathered as soluble protein extracts and preserved at -70 °C for later use.

For each treatment group, 100 oocytes were taken to isolate protein extracts for western Blot detection and pull-down analysis by homogenizing them in 200 μl of extraction buffer with the same method described above for various tissues. Treatment groups included LTE-oocytes without progesterone treatment (Li, Immature oocytes or their protein extracts from toads kept in a low-temperature environment)) and after induced maturation (Lm, Mature oocytes or their protein extracts from toads kept in a low-temperature environment) and HTE-oocytes without (Hi, Oocytes, or protein extracts of oocytes, that have not been subjected to progesterone-stimulation from toads raised in a high-temperature environment) and with progesterone treatment (Hm, Oocytes, or protein extracts of oocytes, that have undergone progesterone-induced maturation from toads raised in a high-temperature environment).

To localize tUCHL1 in oocytes, the nuclei (germinal vesicles, GV) were aspirated out of LTE-oocytes manually with a glass pipette under a stereomicroscope, transferred into 1×SDS protein loading buffer, homogenized, and boiled for 10 min. After centrifugation at 12,000 g for 30 min, the supernatants were collected. These samples were further analyzed as GV protein extract samples. Oocyte cytoplasmic soluble proteins were extracted by centrifugation at 12,000 g as above. The, the precipitates were collected and boiled for 10 min in 1×SDS protein loading buffer. These protein samples were further analyzed as oocyte cytoplasm insoluble samples.

### Immunochemical detection of ovarian and oocyte sections

Ovaries from prepubertal toads with early-stage oocytes were fixed in Bouin's solution for 24 h, and fully developed oocytes isolated from adult toads were fixed with 4% paraformaldehyde in 0.1 mol/L phosphate buffer (pH 7.4) for 24 h. After the routine ethanol gradient dehydration and xylene clearing, the materials were embedded in paraffin, which was cut into 6-μm thick sections for immunochemical analysis of tUCHL1.

The paraffin sections were dewaxed and rehydrated through an alcohol gradient to PBS. Next, the samples were kept in boiling 0.01 M sodium citrate buffer (pH 6.0) for 5 min in a microwave oven 3 times to expose antigens and then immersed in 3% hydrogen peroxide in PBS for 10 min at room temperature to inactivate endogenous peroxidase. To block a non-specific immune reaction, the slides were incubated in blocking solution (10% normal donkey serum in PBS) for 1 h. Incubation with mouse monoclonal antibody against tUCHL1 (prepared in our lab [5], at a final concentration of 2 μg/ml in blocking solution) was conducted at 37 °C for 1 h. After 3 washes of 10 min in PBST (0.05% Tween-20 in PBS), the slides were incubated with biotin-labeled donkey anti-mouse IgG (1:200 dilution, Abcam, USA) at 37 °C for 1 h. After standard washes, streptavidin-labeled peroxidase (Zymed Laboratories Inc.) was used for identification of the presence of the immune complexes. Finally, a DAB chromogenic kit (Liquid DAB-Plus Substrate Kit, Zymed Laboratories Inc.) was used to stain the tUCHL1 immunological complex a brown color in the growing oocytes. For the fully grown oocytes, an AEC kit (Wuhan Boster Bio-engineering Ltd. Company) was applied to stain the UCHL1 immunoreactive signal a red color. Sections were counterstained with hematoxylin, mounted in neutral balsam, and observed and photographed under a microscope (Nikon 50i, Japan). For immunofluorescent staining of the fully grown oocytes section, an Alexa Fluor 488-conjugated goat anti-mouse IgG antibody (1:1000 dilution, Invitrogen, Molecular Probes, USA) was used to recognize the tUCHL1 monoclonal antibody. The sections were then examined and recorded using a fluorescent microscope (Nikon 50i, Japan). To exclude non-specific staining, 16 μg of recombinant GST-tUCH was applied to neutralize 1 μg of primary polyclonal or monoclonal antibody against UCH-L1 as an immunological control. As a molecular weight reference during WB detection, the recombinant tUCHL1 was obtained by cleaving the GST-tUCHL1 with bovine thrombin (Pharmacia Biotech.) as reported[5].

### Spectrofluorimetric detection of UCH activity

Following Dang [10] and Strayhorn [11], Ub-AMC (ubiquitin C-terminal 7-amido-4-methylcoumarin, Calbiochem) was used as the substrate for UCH activity detection. Fluorescence intensity (Fi) at a wavelength of 460 nm (λex = 380 nm) was detected with a fluorescence spectrophotometer (Hitachi 650-10S, Recorder 056), and the change curves over time were recorded. Each 5-μl oocyte soluble protein sample (20 μg) at a concentration of 4 μg/μl was mixed with 242.5 μl of Buffer A (50 mmol/L Hepes-KOH, pH 7.8, supplemented with 0.5 mmol/L EDTA). Fi/time curves were recorded for 3-5 min after incubating the samples at room temperature for 30 min. After 2.5 μl of Ub-AMC (5 μmol/L) was added to reach a final concentration of 50 nmol/L with thorough mixing, we continued to record the Fi/time curves. Recombinant GST-tUCHL1 and active center mutated GST-tUCHL1 (C90S) were used as controls [9].

### Affinity assay and western blot detection

Samples containing 200 μg of soluble protein were diluted individually with extraction buffer into a 200 μl volume. Next, 20 μl of GST-Sepharose 4B suspension, which was prepared in our laboratory, was added to each sample, and then the mixtures were agitated gently on a rotor for 2 h. After centrifugation at 500 g for 3 min, the supernatant was recovered. Then, 20 μl of GST-tUCHL1-Sepharose 4B beads (prepared in our laboratory) was added to each sample, and the mixtures were rotated gently at 4 °C overnight. The GST-tUCHL1-Sepharose 4B beads recovered by centrifugation, as well as the GST-Sepharose 4B precipitates obtained above, were all rinsed in 500 μl of extraction buffer 6 times. The specific adsorption proteins were eluted by boiling in 15 μl of 1×SDS-PAGE loading buffer for 5 min. 

For immunoprecipitation by anti-p34cdc2, 20 µl of mouse monoclonal IgG 2a agarose bead suspension (p34cdc2 of human origin (17) AC, sc-054, Santa Cruz Biotechnology, Inc.) was used to absorb toad homologs of p34cdc2 and conjugated proteins from 120 µg (500 µl) of protein extract. For immunoprecipitation by anti-cyclin B1, 20 µl of mouse monoclonal IgG1 agarose bead suspension (human recombinant cyclin B1 (GNS1) AC, sc-245, Santa Cruz Biotechnology, Inc.) was applied to isolated tUCHL1. Proteins in the immunoprecipitates were eluted in 1×SDS loading buffer as described above for western blot detection of tUCHL1 with polyclonal antibody against human UCHL1 (Chemicon international Inc.). 

For the affinity assay with p13suc1-agarose beads (Oncogene), the protein extracts first were subjected to Sepharose CL-6B pre-adsorption, and then 20 μl of p13suc1-agarose beads were used to isolate the proteins bound with p13suc1. Next, the four types of protein extract samples (Lm, Li, Hm, and Hi) were incubated at 37 °C for 30 min in extraction buffer supplemented by the final addition of 1 mM ATP, 80 μg/ml creatine kinase (Roche) and 10 mM creatine phosphate (Roche) according to Jessus' method [12] to determine the phosphorylation in vitro, which is referred to as the ATP regenerating system hereafter. Proteins in the affinity complex were eluted in 1×SDS loading buffer as described above for western blot detection of tUCHL1 with monoclonal antibody (prepared in our lab).

The protein elution samples in 1×SDS loading buffer were cleared by a brief centrifugation. All of the protein samples were separated using 12% sodium dodecyl sulfate polyacrylamide gel electrophoresis (SDS/PAGE), and the protein bands were electro-transferred by a wet method onto the nitrocellulose blotting membrane (Millipore, USA). The nitrocellulose membrane with the western blots was immersed in 7% skim milk in TBST (25 mmol/L Tris-HCl pH 8.0, 150 mmol/L NaCl, 2.7 mmol/L KCl, 0.05% Tween-20) to block non-specific binding and maintained at room temperature for 1 h before western immunoblot detection. 

**Figure 1:**
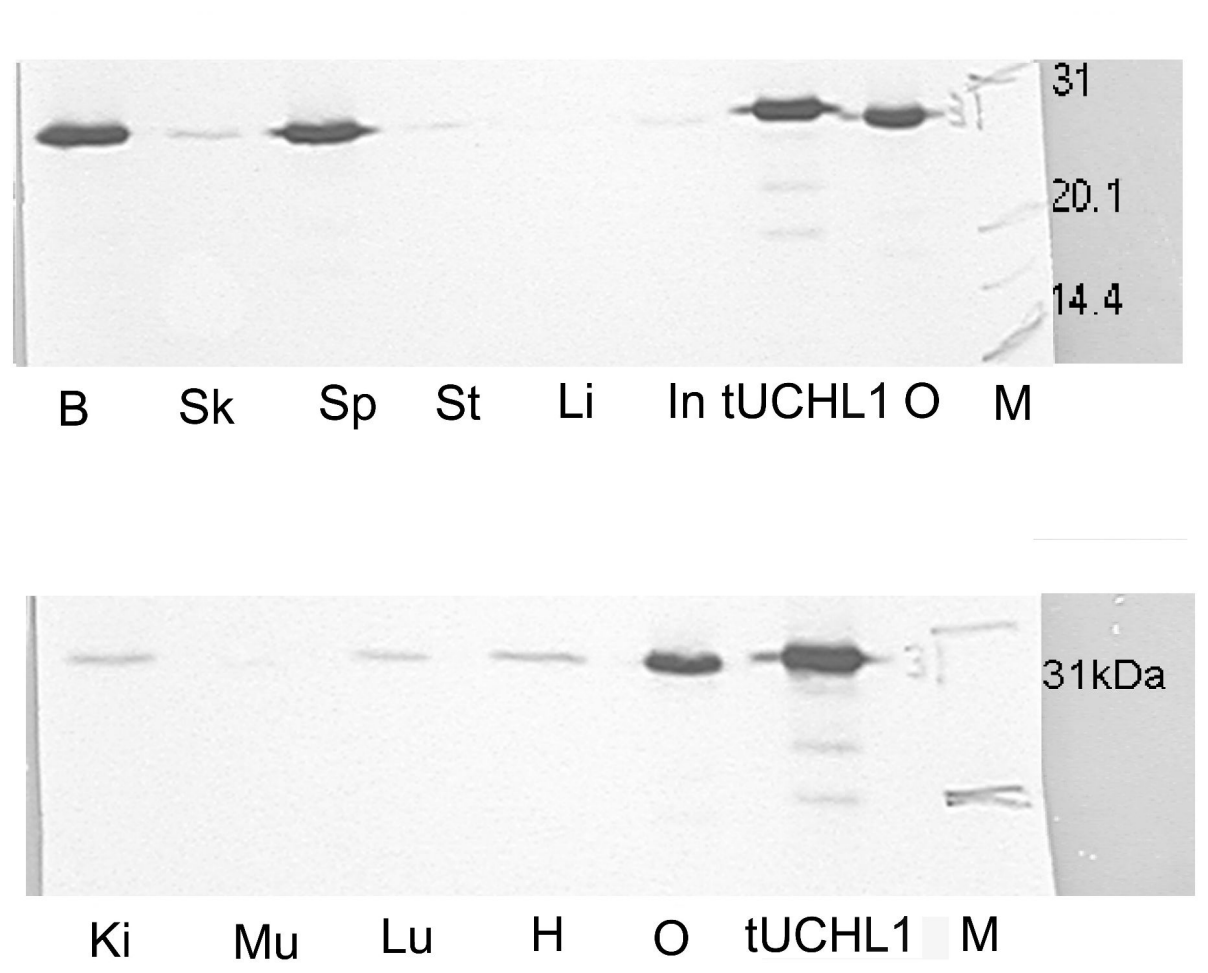
Tissue-specific expression of tUCHL1 in toads. The soluble protein extracts were separated by 12% SDS-PAGE, and the protein bands were identified using mouse monoclonal antibody against tUCHL1, colored with DAB on the nitrocellulose membrane. In each lane, 20 μg of soluble protein extract from various tissues was loaded separately, except for 0.5 μg of recombinant protein as a control. The abbreviations are as follows: B: brain; Sk: skin; Sp: spermary; St: stomach; Li: liver; In: intestine; tUCHL1: Recombinant tUCHL1 expressed in *E. coli* (DE3), which has a molecular weight of approximately 29 kDa; O: oocyte; Ki: Kidney; Mu: muscle; Lu: lung; H: heart; M: marker. The experiment was independently repeated twice.

For western blot detection of tUCHL1, p34cdc2 and cyclin B1, the blots were incubated for 1 h with anti-tUCHL1 antibody at a final concentration of 2 μg/ml in blocking solution, with anti-human p34cdc2 rabbit polyclonal antibody (ABR. Affinity Bioreagents, USA) and anti-human cyclin B1 rabbit polyclonal antibody (cyclin B1 (H-433): sc-752, Santa Cruz Biotechnology, Inc. USA), both at a dilution of 1/200 in blocking solution. As a loading control, beta-actin was detected with a rabbit polyclonal antibody (Cell Signaling Technology, Ltd. Inc., USA) at a 1:1000 dilution. After being washed in TBST for 10 min 3 times, the blots were incubated with alkaline phosphatase (AP) conjugated goat anti-mouse IgG (in 1/500 dilution, Zymed Lab. Inc.), followed by the regular washing procedure. AP-marked immune complexes were stained on the blotting membrane using a BCIP/NBT Kit (Zymed Lab. Inc.), as shown in Figure 1, or visualized through a chemiluminescence reaction for 3 min with a PhosphaGLO™ AP Substrate kit (KPL, Gaithersburg, MD, USA) according to the manufacturer’s protocol. To compare the relative contents between Lm, Li, Hm and Hi, the Tanon Gis program (Shanghai Bio-Tech, Shanghai) was used to evaluate the protein band intensity of p34cdc2, cyclin B1, cyclin B1p, and tUCHL1 with beta-actin as a reference. The relative quantitative values of protein bands were analyzed with the SPSS 18.0 software (SPSS Inc., Chicago, IL, USA). The differences between groups were tested for significance using the independent sample t-test. Statistical significance was set at p < 0.05.

## Results

### Tissue-specific expression of tUCHL1

The western blot results revealed that tUCHL1 was detected as a single band with an apparent molecular weight of approximately 28 kDa. The tUCHL1 protein was determined to be very abundant in the brain, testes and oocyte samples; scarce in the skin, stomach, lung, kidney, small intestine and heart samples; and nearly undetectable in the liver and muscle samples (Figure 1). 

### Immunological localization of tUCHL1 in toad oocytes at different stages

**Figure 2:**
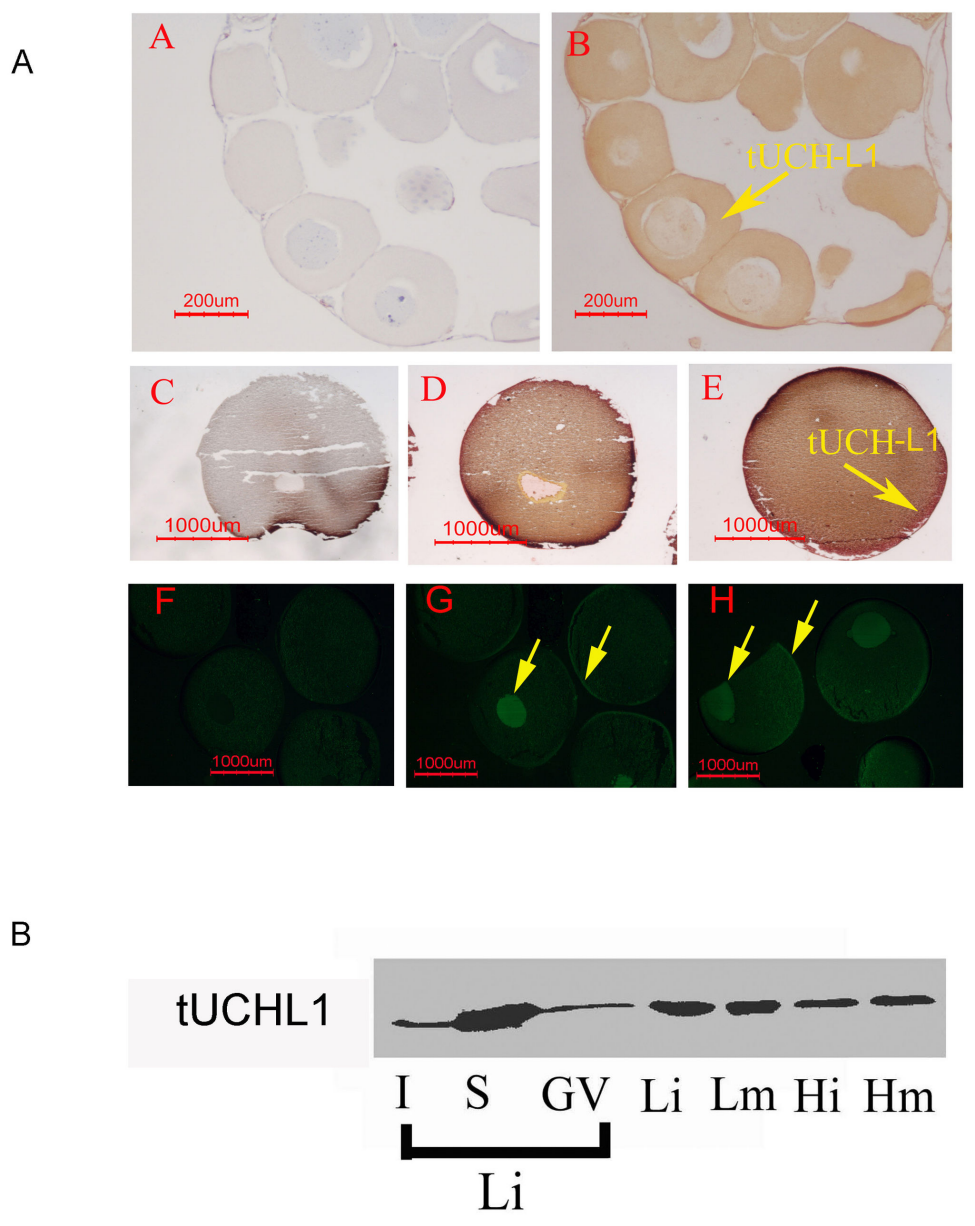
Immunological localization of tUCHL1 in toad oocytes. A: Control for immunological detection in B with primary antibody blocking. B: Immunohistochemical detection of tUCHL1 on sections of a juvenile toad's ovary, visualized in brown using a DAB kit. C: Control for immunological detection in D and E with primary antibody blocking. D: tUCHL1 immunological detection in immature LTE-oocytes (Li) at stage IV, colored red with an AEC kit. E: tUCHL1 detection in mature oocytes (Lm) in parallel with D. F: control for immunofluorescent detection in G and H with primary antibody blocking. G: Immunofluorescence of tUCHL1 in HTE-oocytes without progesterone treatment (Hi). H: tUCHL1 immunofluorescence detection in immature LTE-oocytes (Li) in parallel with G. Yellow arrows indicate the distribution of tUCHL1. B: Western blotting analysis of tUCHL1 in different parts of toad oocytes. Lane I: 1/10 volume of the insoluble protein part derived from 40 LTE-oocytes; S: 1/10 volume of the soluble protein part derived from 40 LTE-oocytes; GV: protein extract derived from 10 nuclei isolated from LTE-oocytes; Li: soluble protein extract from 2 immature LTE-oocytes; Lm: soluble protein extract from 2 mature LTE-oocytes; Hi: soluble protein extract from 2 HTE-oocytes without progesterone treatment; Hm: soluble protein extract from 2 progesterone treated HTE-oocytes. All the bands in Panel B were visualized by chemiluminescence. Each experiment was repeated twice independently.

In the growing oocytes (stages I and II), strong tUCHL1 immunoreaction positive signals were present and distributed uniformly over the cytoplasm, and weak tUCHL1 staining occurred in the nucleus (Figures 2A and B). However, in full-grown oocytes (stage IV), tUCHL1 was concentrated in the plasma membrane and the nucleus and present at a lower density throughout the cytoplasm (Figure 2D). In the IHC control (Figure 2C), no positive red signal was found. After maturation with GV breakdown, no dense concentration of tUCHL1 was observed in the equivalent nuclear position, but a high abundance remained in the plasma membrane (Figure 2E). Based on the immunofluorescent staining, in both the HTE-oocytes (Figure 2G) and LTE-oocytes (Figure 2H) but not the control (Figure 2F), tUCHL1 was observed at a higher density in the nucleus and plasma membrane than in the cytoplasm. With careful observation, in HTE-oocytes, nuclear staining was observed to be more intense, whereas in LTE-oocytes, stronger fluorescence appeared in the plasma membrane.

A large amount of tUCHL1 was present in the cytoplasmic soluble protein extracts from full-grown oocytes (Figure 2B, lane S, equivalent to 4 oocyte cytoplasm volumes), whereas a small amount of tUCHL1 was observed in the corresponding insoluble precipitation in parallel to lane S (Figure 2B, lane I, equivalent to 4 oocyte cytoplasm volumes). In addition, a small amount of tUCHL1 was also located in the germinal vesicle (Figure 2B, lane GV, 10 germinal vesicles). The germinal vesicle could not be manually separated in the HTE-oocytes. Thus, here only the results for the LTE-oocytes are shown. The amount of tUCHL1 in toad oocyte protein extracts from the Lm and Li groups was greater than that from the Hm and Hi groups (Figure 2B, lanes Li, Lm, Hi, and Hm, each from 2 oocytes).

### Comparisons of MPF and tUCHL1 between the high and low temperature oocytes

**Figure 3:**
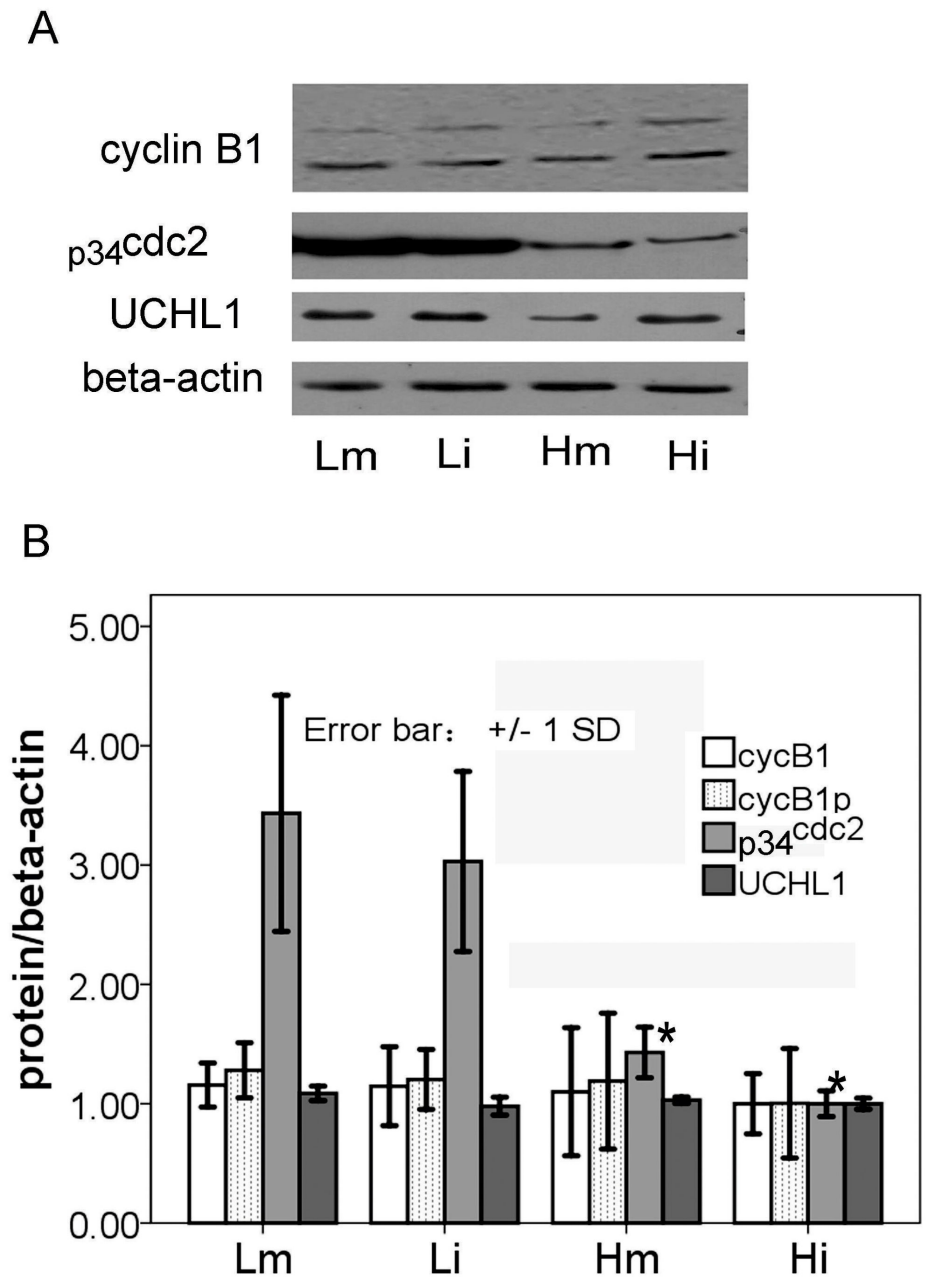
The protein quantification assays of tUCHL1, p34^cdc2^ and cyclin B1 in four different oocyte protein extracts. The protein amounts of tUCHL1 and MPF subunits (p34^cdc2^ and cyclin B1) in the four types of oocyte protein extracts were evaluated in reference to beta-actin. The total soluble protein extracts of 20 µg individually from Lm, Li, Hm and Hi samples were used to detect tUCHL1, p34^cdc2^ and cyclin B1 by the western Blotting method, and here, all the bands were visualized by chemiluminescence. Lm and Li indicate immature or mature LTE-oocytes; Hm and Hi indicate THE-oocytes with or without progesterone treatment. Panel A presents a typical picture of western Blotting bands among three independent experiments. Panel B shows the optical density values from three independent western Blotting results. The longitudinal axis indicates ratios between the optical density values of the denoted proteins and those of beta-actin. According to t-test analysis, only p34^cdc2^ among the denoted proteins was significantly different in its relative values between Lm and Hm (p=0.027), or Li and Hi (p=0.010). A significant level less than 0.05 is marked at the tops of corresponding bars of Hm and Hi with an asterisk.

While polyclonal antibodies were used to target two subunits of MPF, p34cdc2 and cyclin B1, p34cdc2 homologous proteins in toad oocytes were detected by WB in one targeted band, whereas cyclin B1 homologous proteins were detected in 2 targeted bands with a molecular weight range of 55-60 kDa. These two bands were provisionally identified as cyclin B1 (cycB1) and its phosphorylated form (cycB1p). For the detection of UCHL1, mouse monoclonal antibody was applied, and only one 28 kDa band was generated. The amount of p34cdc2, but not cyclin B1 or tUCHL1, displayed significant differences in total soluble protein between high-temperature and low-temperature oocytes (Figure 3).

### tUCHL1 activity in low- and high-temperature oocytes

**Figure 4:**
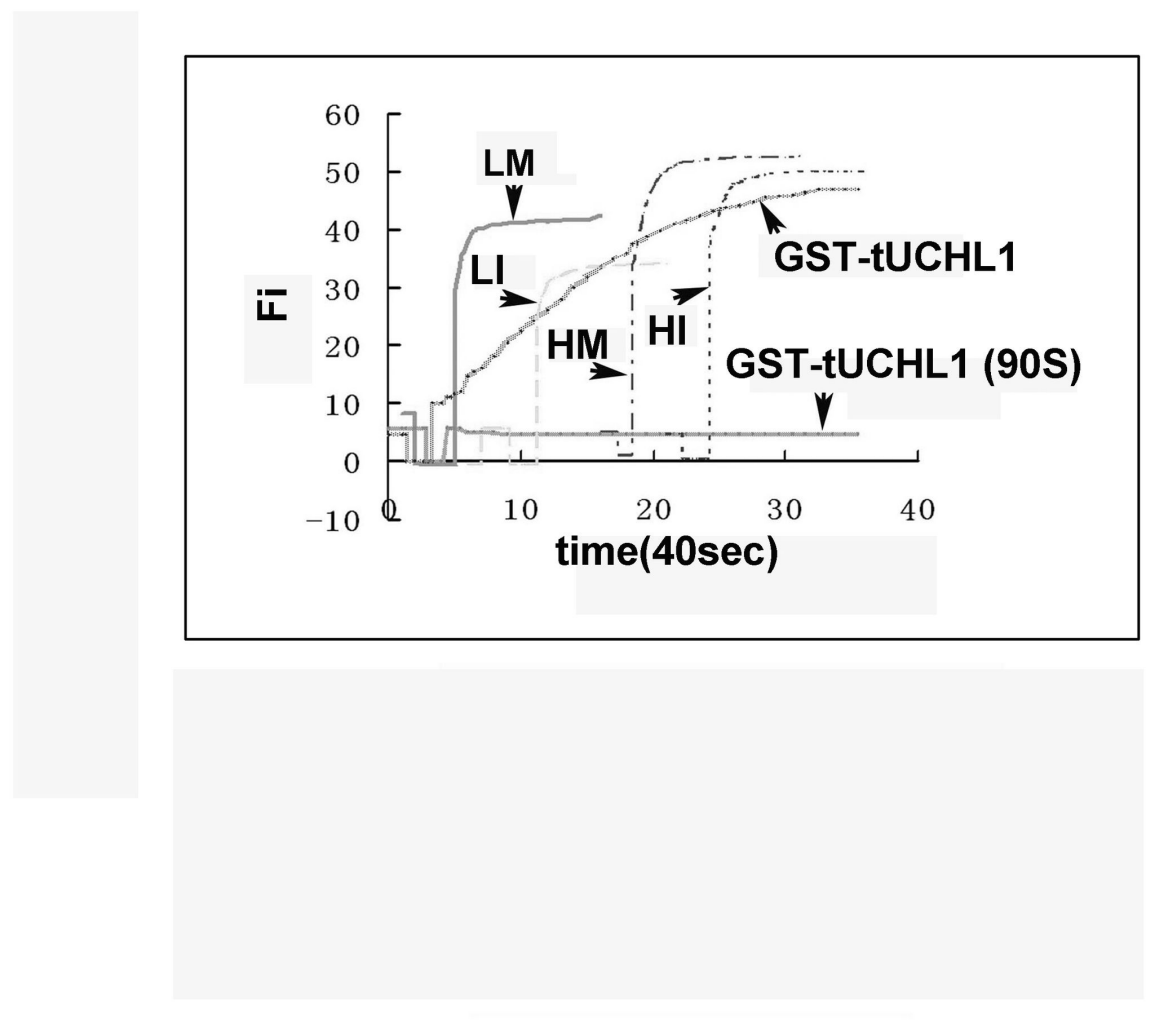
The comparisons of UCH activity of tUCHL1 between LTE-oocytes and HTE-oocytes. A: ubiquitin carboxyl-terminal hydrolase (UCH) activity of four types of oocyte protein extracts. Lm: mature LTE-oocytes; Li: immature LTE-oocytes; Hm: progesterone treated HTE-oocytes; Hi: HTE-oocytes without progesterone treatment. Oocyte soluble protein extracts were prepared at the final concentration of 20 µg/ml, and the two types of recombinant proteins are both used at the concentration of 20 nmol/L. The sharp drops in the curves at the beginning were caused by the light path closure to add reaction substrate Ub-AMC. The experiment was repeated twice independently, and the recombinant tUCHL1 was applied at a relative low concentration to obtain an appropriate slope. To draw all the curves in the same diagram, their starting points were adjusted.

Oocyte soluble protein extract containing tUCHL1 can catalyze the hydrolysis of Ub-AMC to generate free AMC, which produces fluorescence of a specific wavelength. The fluorescence intensity, abbreviated as Fi hereafter, reflects the concentration of AMC, denoted as [AMC], which is expressed in arbitrary units here as elsewhere [9]. For the balanced top values in the Fi/time curves of the catalytic reactions, the experimental groups can be listed in decreasing order as Hm, Hi, Lm and Li, with the same oocyte soluble protein concentrations of 20 μg/ml (Figure 4). There was no obvious difference among groups in the curve slopes reflecting UCH hydrolase activity. As for the controls, with both recombinant proteins at a final concentration of 20 nmol/L, the Fi/time curve of recombinant GST-tUCHL1 had an obviously smaller slope, whereas the curve of recombinant GST-tUCHL1 (90S) with a mutation at the active center had no rise, indicating that no catalytic reaction occurred to generate [AMC].

### tUCHL1 was demonstrated to be associated with p34^cdc2^ by in vitro binding analysis

**Figure 5:**
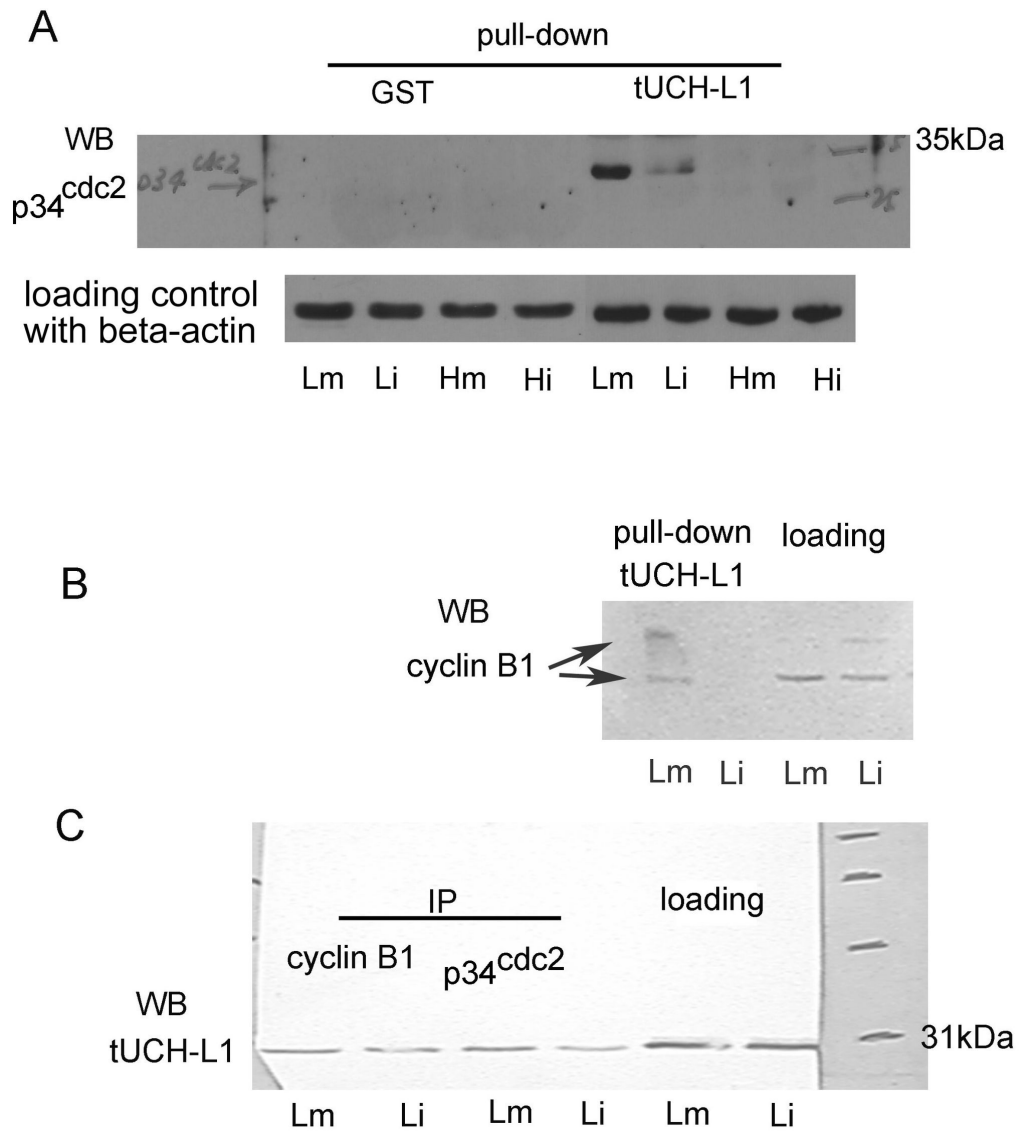
tUCHL1 binds to MPF components p34^cdc2^ or cyclin B1 in different oocyte protein extracts. Panel **A**: A pull-down analysis (specific adsorption) with recombinant GST-tUCHL1 and with GST as a control, which were coupled to Sepharose 4B beads, respectively, from 200 µg of Lm, Li, Hm, Hi oocyte soluble protein extracts, was performed for **p34^cdc2^** assay by western Blotting analysis with beta-actin detection as a pull-down sample loading control. Panel B: Western blotting analysis for cyclin B1 after pull-down analysis with GST-tUCHL1 from oocyte protein extracts of Lm and Li, with 20 µg of total protein extracts (Lm and Li) as loading control was performed. Panel C: The results of immunoprecipitation (IP) with mouse monoclonal antibodies against **p34^cdc2^** and cyclin B1 from 120 µg of toad oocyte protein extracts are shown. Isolated protein complexes were then detected by western blotting analysis for tUCHL1. Loading denotes the lanes with 15 µg of total protein extracts as loading control. Lm and Li indicate immature or mature LTE-oocytes; Hm and Hi indicate THE-oocytes with or without progesterone treatment, respectively.

Recombinant GST-tUCHL1 could separate the MPF subunit from the protein extracts of LTE-oocytes (Figure 5A, Lm and Li) but not those of HTE-oocytes. Moreover, the binding that occurred in LTE-oocyte protein extracts was specific to p34cdc2, as no binding to GST was detected. Its band corresponded to a molecular weight of approximately 34 kDa and not two or three different phosphorylated bands as usual. In addition to the lack of binding detected in the HTE-oocyte extract, the quantity of p34cdc2 isolated from the mature LTE-oocyte extract was much larger than from the immature extract. 

Cyclin B1 was detected as two protein bands in the oocyte cytoplasmic soluble protein extracts of the Lm and Li groups; however, in Lm group only, it could be separated by tUCHL1 pull-down as described above (Figure 5B). 

Based on immunoprecipitation, tUCHL1 could be bound to both cyclin B1 and p34cdc2 in the Lm and Li oocyte protein extracts. Moreover, Lm oocyte protein extracts exhibited a greater amount of tUCHL1 adsorption than the Li group (Figure 5C).

**Figure 6:**
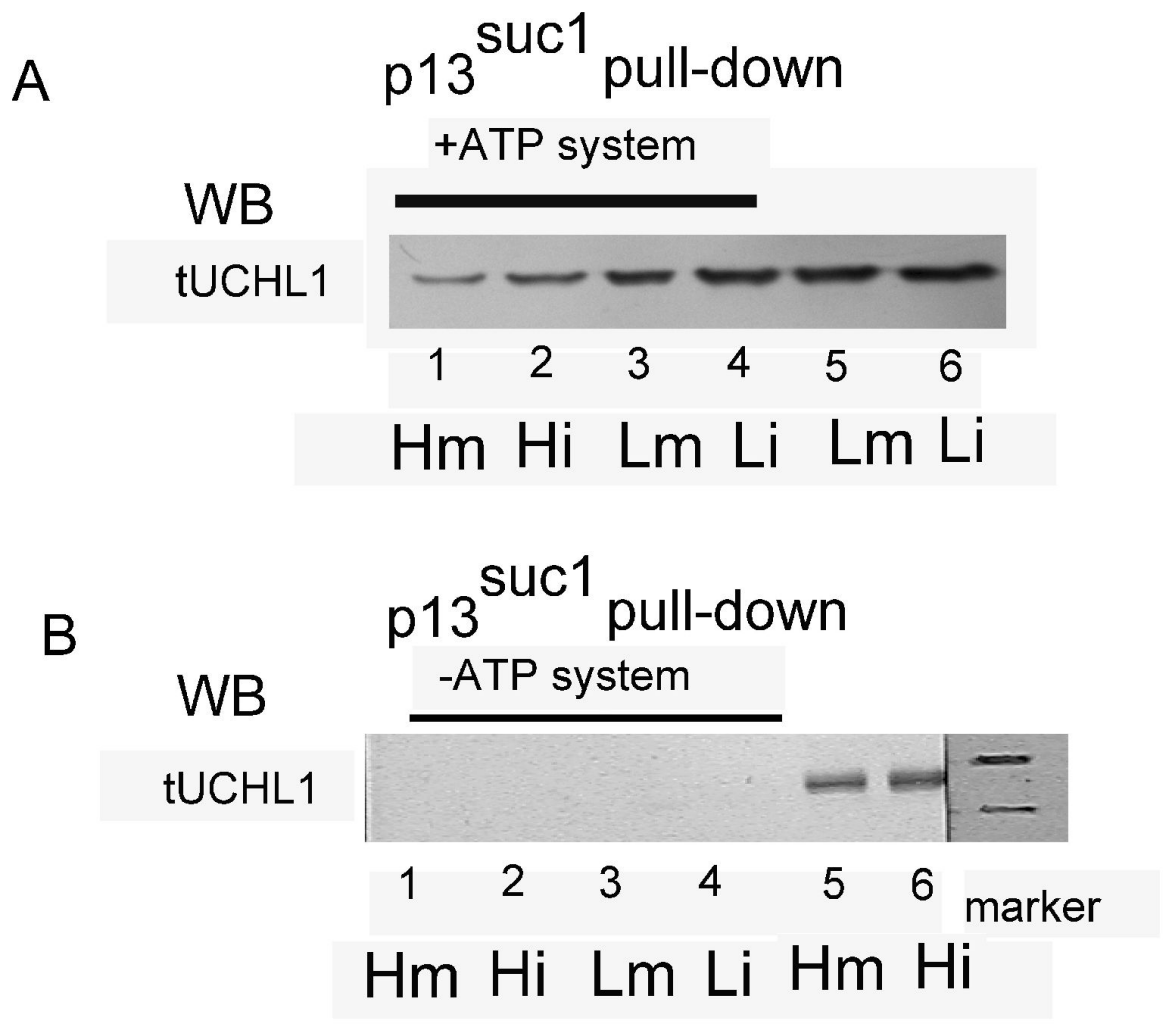
Pull-down analysis with recombinant p13^suc1^ for tUCHL1 isolation from oocyte protein extracts. Panel A: After toad oocyte protein 500 µg extracts were treated with the ATP system, and affinity adsorption (pull-down) was performed with recombinant p13^suc1^-argrose to isolate protein complex from the oocyte protein extracts. The isolated protein complex was used to detect tUCHL1 by western Blotting analysis. Lanes 1-4 show results of the pull-down, and lanes 5-6 contained two loading controls with 20 μg of total protein extracts. B: Affinity adsorption results without the ATP system treatment run in parallel to the lanes in panel A. Lanes 1-4 are the corresponding adsorption results, and lane 5-6 indicate two other loading controls than those used in panel A. Lm and Li indicate immature or mature LTE-oocytes; Hm and Hi indicate THE-oocytes with or without progesterone treatment, respectively.

Via pull-down analysis with p13 conjugated agarose, p13suc1 was determined to bind specifically with tUCHL1 in oocyte protein extracts under the ATP system, similar to the Lm, Li, Hm and Hi groups (Figure 6A), whereas tUCHL1 protein could not be isolated by p13suc1 if the ATP system had been omitted (Figure 6B). The tUCHL1 protein content estimated by pull-down did not exhibit significant differences between HTE-oocytes and LTE-oocytes. Compared with the loading control, no electrophoretic shift of tUCHL1 bands occurred, even with the ATP system treatment. 

## Discussion

### Conservation of tUCHL1 in evolution

Evolutionary conservation of UCHL1 has occurred in terms of its amino acid composition and structure as well as its distribution in animal tissues, including the specific distribution and activity of UCH in oocytes. The amino acid sequence of toad UCHL1 protein (gi|11321437) shows significant homologies to mouse UCHL1 (gi|148,705,826) (58 % similarity) and similar proteins in the zebrafish (gi|37,722,017) (51 % similarity); *Drosophila melanogaster* (gi|17,136,836) (39 % similarity); and rice (gi|125,591,179) (34 % similarity). Based on its presence in frogs, trout, chickens, guinea pigs, rabbits, sheep, goats, cattle and human brain extracts, it can be hypothesized that UCHL1 protein evolved at least 400 million years ago and has been highly conserved throughout evolution [13]. The distribution of tUCHL1 in different toad tissues is also found to be highly conserved in the case of higher vertebrates, such as in mammals. UCHL1 exhibits highly specific expression levels in the mouse brain and gonads and significantly lower levels in other tissues [8,14,15].

Our results demonstrated that tUCHL1 is primarily located in the cytoplasm of toad oocytes at different developmental stages. In agreement with a previous report that UCHL1 can enter the nucleus of cancer cells [16], signals of tUCHL1 were detected by immunohistochemistry and immunofluorescence in the nucleus of toad oocytes at different developmental stages. In mammals, UCHL1 is concentrated in the cortex of mouse oocytes[17], with a similar distribution pattern to p34cdc2 [18] in *Xenopus* oocytes, so we speculate that the connection of UCHL1 with p34cdc2, as we obtained in toad oocytes above, was involved in the mechanisms of oocyte maturation. Because it was thought concentrated in the plasma membrane, its role in the prevention of polyspermic fertilization was also suggested [19–22]. Great differences were found between LTE-toad and HTE-toad oocytes, that is, LTE-oocytes achieved maturation competence, whereas HTE-oocytes did not [2]. Owing to more intense tUCHL1 immunostaining in the nucleus of HTE-toad oocytes than in LTE-oocytes, we hypothesized that an increased tUCHL1 concentration in the nucleus may be implicated in oocyte maturation incompetence. And Western blot result on LTE-oocytes supported that the tUCHL1 was present in the GV part although the sampling contamination from cytoplasms could not be totally excluded. However, we were not able to provide Western blot data on THE-oocytes to support our above hypothesis on tUCHL1 because, unlike in LTE-oocytes, it was very difficult to separate the germinal vesicles of HTE-oocytes manually. Certainly, otherwise the stronger connection of the germinal vesicle to the cytoplasmic components in HTE-oocytes compared to LTE-oocytes also suggested other mechanisms to explain for the maturation incompetence caused by the high temperature environmental conditions.

### Differences in MPF and tUCHL1 protein between HTE-oocytes and LTE-oocytes

Relative to beta-actin, as an MPF subunit, p34cdc2 exhibited a significant difference in HTE-oocyte and LTE-oocyte protein extracts, whereas cyclin B1 did not. These results suggest that low temperatures during winter hibernation contribute to oocyte maturation competence via the accumulation of p34cdc2 protein in oocytes. Using the unilateral cryptorchid model, it was determined that rabbit abdominal temperature had a significant influence on the translation of p34cdc2, resulting in a decrease in its protein content in pachytene/diplotene primary spermatocytes [23]. Thus, we hypothesized that either in spermatogenesis or in oogenesis, temperature could affect the intracellular p34cdc2 content through some unknown mechanism. As a special example, the influence of temperature on p34cdc2 content could underlie the dependence of toad oocyte maturation competence on winter hibernation.

With GST-tUCH and GST-tUCH (90S) as controls, the reaction curve slopes of Lm, Li, Hm, and Hi are similar (Figure 4), suggesting that UCH enzyme activity has no significant differences among the groups. No obvious differences in tUCHL1 protein content or UCHL1 enzyme activity could be observed between HTE-oocyte and LTE-oocyte protein extracts. Thus, tUCHL1 may not contribute to the low-temperature dependence of toad oocyte maturation competence. The higher balance top points of the Fi/time curves for HTE-oocytes suggested that they had a smaller free ubiquitin pool than in the LTE-oocyte protein extracts, as increased endogenous levels of free ubiquitin could move the UCH enzyme-catalyzed reaction balance to the left. Considering that the size of the HTE-oocytes (diameter: 1642 ± 12 µm) was smaller than that of the LTE-oocytes (diameter: 1707 ± 26 µm), we compared the content of tUCHL1 in a certain number of oocytes and determined that its level was slightly lower in HTE-oocytes, but we do not believe that this difference is the mechanism for the high-temperature-driven maturation potential loss because even individual LTE-oocytes with smaller diameters have normal maturation potential (related data were not included in this paper). Thus, we confirmed a contribution from p34cdc2 protein quantity difference between LTE-oocytes and THE-oocytes. In addition, we excluded possible differences in tUCHL1 protein levels as s possible reason. Considering the differences of candidate protein for tUCHL1 reported by Lu [4], we deduced that the previous candidate was not tUCHL1 itself. Therefore, the candidate identity requires further verification.

### The association between tUCHL1 and MPF

Recombinant GST-tUCHL1 could adsorb cyclin B1 and p34cdc2 proteins. Conversely, UCHL1 was bound during immune precipitation of monoclonal antibodies against human p34cdc2 and cyclin B1. Moreover, p13suc1 could bind to p34cdc2 [24], and we found in this paper that recombinant p13suc1-agarose could adsorb tUCHL1 protein from oocyte protein extracts after treatment with an ATP regenerating system. In that situation, we hypothesized that p13suc1 separated tUCHL1 indirectly, possibly via p34cdc2. Our results support that tUCHL1 binds to MPF in toad oocytes, but the binding was not dependent on the modifications of tUCHL1, cyclin B1 and p34cdc2 such as ubiquitination or phosphorylation because no electrophoretic shift was detected by western blot analysis. Considering that cyclin B1 was separated by tUCHL1 only from the mature LTE-oocyte protein extract, it was deduced that tUCHL1 binds with p34cdc2 and indirectly absorbs cyclin B1 via p34cdc2, possibly because p34cdc2 and cyclin B1 combine into MPF more in mature oocytes than in immature oocytes, as in the case of the frog *Rana japonica* [25].

The protein p34cdc2 was only isolated from LTE-oocyte protein extracts by GST-tUCHL1 pull-down, and the quantity obtained from mature LTE-oocyte protein extract was much greater than that from immature LTE-oocyte protein extract. Considering that p34cdc2 content was more abundant in LTE-oocytes than in HTE-oocytes relative to beta-actin, we believed that the absent detection of protein p34cdc2 in binding to recombinant tUCHL1 from HTE-oocyte protein extracts may have been because of its relatively low protein content in HTE-oocytes caused by high environmental temperatures. In addition, though the amount of p34 bound to the recombinant tUCHL1 was significantly different between the mature and immature LTE-oocytes, some other molecules could have played a role in interfering with the binding of p34 to tUCHL1, as p34cdc2 protein content was similar between mature and immature oocytes. As for the binding with cyclin B1, the complex of p34cdc2 and cyclin B1, or MPF, existed in LTE-oocytes whether they were mature or not, as detected in the case of *Xenopus* [26,27], but the quantity of the complex is much lower in immature oocytes and mainly exists as free p34cdc2 and cyclin as in frogs [25,28]. Therefore, we could not detect cyclin B1 even with a small quantity of p34cdc2 isolated from the immature oocyte protein extract. Conversely, and interestingly, endogenous tUCHL1 could be specifically absorbed during precipitation with both mature and immature oocyte extracts, though the amount of absorbed tUCHL1 was a slightly larger than in the immature oocyte protein extracts. As for the fact that the quantity difference between Lm and Li of natural tUCHL1 isolated by p34cdc2 or cyclin B1 antibodies was not as large as that of p34cdc2 or cyclin B1 in GST-tUCHL1 pull-down detection, we speculate that the amount of natural tUCHL1 in oocytes was relatively large, and, accordingly, it was more easily isolated by the immune precipitation against p34cdc2 or cyclin B1, resulting in less difference between Lm and Li.

Turning to our adsorption test results with p13suc1 as bait wherein tUCHL1 was isolated by affinity adsorption of p13suc1 and it depended on the phosphorylation of the ATP regeneration system, it is possible that tUCHL1 bound only the activated form of p34cdc2 with a modification change similar to the phosphorylation of the *Xenopus* 161 site [29,30] and did not bind inactive forms. However, we could not detect its corresponding electrophoretic shift with western blotting. Therefore, it is more likely that there were other factors involved in the dependence on phosphorylation to mediate the binding of p34cdc2 and tUCHL1. A comprehensive analysis on all affinity analysis results (Figures 5 and 6) indicates that the tUCHL1 isolation of p34cdc2 from oocyte extracts was dependent on a low-temperature environment, but the indirect combination of p13suc1 and tUCHL1 only relied on the ATP system treatment. These results require further experiments for confirmation.

In conclusion, this study revises the established understanding of the mechanism by which temperature affects toad oocyte maturation. Although it was once believed that the tUCHL1 candidate content was affected by a high-temperature environment, in the present research, we excluded the involvement of the tUCHL1 level and UCH enzyme activity in the loss of maturation competence caused by high temperatures. However, we unexpectedly discovered that the specific binding of tUCHL1 and MPF component p34cdc2 may be involved in the mechanism in which tUCHL1 is necessary for GVBD of toad oocytes (18). In addition, we determined that the oocyte content of p34cdc2 was significantly reduced as a result of high temperatures, which may be the key mechanism driving the established understanding that the maturation competence of toad oocytes is low-temperature dependent.
